# The PI3K/Akt-Nrf2 Signaling Pathway and Mitophagy Synergistically Mediate Hydroxytyrosol to Alleviate Intestinal Oxidative Damage

**DOI:** 10.7150/ijbs.97263

**Published:** 2024-08-06

**Authors:** Xiaobin Wen, Shanlong Tang, Fan Wan, Ruqing Zhong, Liang Chen, Hongfu Zhang

**Affiliations:** State Key Laboratory of Animal Nutrition and Feeding, Institute of Animal Sciences, Chinese Academy of Agricultural Sciences, Beijing, 100193, China.

**Keywords:** Oxidative stress, Nrf2 signaling pathway, Mitophagy, Hydroxytyrosol, Intestinal health

## Abstract

Oxidative stress is a major pathogenic factor in many intestinal diseases, such as inflammatory bowel disease (IBD) and colorectal cancer (CRC). The Nrf2 signaling pathway and mitophagy can reduce reactive oxygen species (ROS) and alleviate oxidative stress, but their relationship is unclear. Hydroxytyrosol (HT), a polyphenolic compound abundant in olive oil, has strong antioxidant activity and may help treat these diseases. We used pigs as a model to investigate HT's effect on intestinal oxidative damage and its mechanisms. Diquat (DQ) induced oxidative stress and impaired intestinal barrier function, which HT mitigated. Mechanistic studies in IPEC-J2 cells showed that HT protected against oxidative damage by activating the PI3K/Akt-Nrf2 signaling pathway and promoting mitophagy. Our study highlighted the synergistic relationship between Nrf2 and mitophagy in mediating HT's antioxidant effects. Inhibition studies confirmed that disrupting either pathway compromised HT's protective effects. Maintaining redox balance through Nrf2 and mitophagy is important for eliminating excess ROS. Nrf2 increases antioxidant enzymes to clear existing ROS, while mitophagy removes damaged mitochondria and reduces ROS generation. This study demonstrates that these pathways collaboratively modulate the antioxidant effects of HT, with neither being dispensable. Targeting Nrf2 and mitophagy could be a promising strategy for treating oxidative stress-related intestinal diseases, with HT as a potential treatment.

## 1. Introduction

Oxidative stress has been widely recognized as one of the major pathogenic factors in various intestinal diseases, including inflammatory bowel disease (IBD) and colorectal cancer (CRC) 1, 2. It primarily compromises intestinal health by causing cell damage, inflammation, and intestinal dysfunction. The excessive production of reactive oxygen species (ROS) and an imbalance in antioxidant defense systems are the main causes of oxidative stress. Excessive ROS can disrupt cellular proteins, lipids, and DNA, leading to fatal cellular damage, among other abnormal reactions 3, 4. Therefore, regulating and reducing ROS levels is a major research focus in mitigating oxidative stress. Intestinal cells are one of the primary types of cells exposed to the external environment within the human body and play a vital role in nutrient absorption and metabolism. Given its unique physiological structure and functional orientation, the intestine is particularly susceptible to external factors, including those that cause oxidative stress. Therefore, regulating oxidative stress has become an important strategy for preventing and treating intestinal diseases. Measures such as searching for and developing antioxidants, optimizing drug treatments, and adjusting lifestyle factors can help alleviate oxidative stress, reducing intestinal disease risk.

The Mediterranean diet is considered one of the healthiest dietary patterns. Numerous studies have revealed that the local population has a lower incidence of diseases such as CRC, cardiovascular disease, and inflammation 5, 6. This is primarily attributed to the crucial component of the Mediterranean diet - olive oil, which is rich in hydroxytyrosol (HT) and offers significant health advantages 7, 8. Hydroxytyrosol (HT), a natural polyphenolic compound, is renowned for its potent antioxidant properties, which enable it to scavenge ROS and reduce the oxidative stress caused by them in the body 9, 10. Notably, oxidative stress plays a significant role in the development and progression of intestinal diseases. Therefore, considering the antioxidant properties of HT, it is reasonable to speculate that HT may have the potential to improve related intestinal diseases, especially in alleviating intestinal oxidative stress. Some research findings have supported this notion. In DSS-induced ulcerative colitis models, HT has been shown to enhance antioxidant enzyme activity, thereby reducing disease activity index and mortality 11. Moreover, HT helps regulate gut microbial balance, maintain gut microbial ecological stability, and promote intestinal health 12, 13. However, the regulatory mechanism of HT has not yet been fully elucidated, which greatly limits our understanding and application of its therapeutic potential in gastrointestinal disease.

Maintaining the physiological level of ROS is crucial for the normal functioning of physiological processes such as immune response, cell signaling transduction, and metabolism 14. To sustain intracellular oxidative balance, the Nrf2 signaling pathway and mitophagy mechanism each play a pivotal role. The Nrf2 signaling pathway is particularly significant in inducing the antioxidant response and has accordingly garnered considerable attention from researchers in the field. Nrf2 is a transcription factor that mainly regulates the transcription of antioxidant genes, such as CAT, SOD, HO-1, and NQO1, by adjusting the expression of antioxidant response elements (ARE), scavenging ROS, achieving defense against cell oxidative stress 15. When Nrf2 expression increases, the expression of antioxidant genes in cells is upregulated, resulting in an improved ability to resist oxidative stress. Our previous research showed that HT could improve oxidative stress in mice by activating the Nrf2 signaling pathway 13. As we all know, mitochondria are the primary production site and the main target of ROS 16. Upon exposure to external harmful stimuli, the damaged mitochondria produce a large amount of ROS, which further exacerbates oxidative damage 17. Mitophagy is a process that helps eliminate potential sources of oxidative stress by engulfing damaged mitochondria and degrading them. Through the selective clearance of damaged mitochondria, mitophagy reduces the production of ROS, thereby protecting cells from oxidative damage 16. The PINK1-Parkin pathway plays an essential role in mitophagy. Parkin is a mitochondrial outer membrane protein with Ser/Thr protein kinase activity and acts as a molecular sensor for damaged mitochondria. PINK1 is a protein with E3 ubiquitin-protein ligase activity, and both participate in the sensing and selective removal of damaged mitochondria 16, 18. Studies have shown that HT could alleviate liver fat deposition and improve mitochondrial function in fish by activating the AMPK/PINK1-mediated mitophagy, thereby regulating lipid metabolism, and providing a potential therapeutic strategy for the prevention and treatment of NAFLD 19, 20. Therefore, we emphasize the interaction between the Nrf2 signaling pathway and mitophagy is particularly crucial in the cellular antioxidant defense mechanism. Nrf2 reduces oxidative stress by regulating the expression of antioxidant genes, while mitophagy prevents the occurrence of oxidative stress by clearing damaged mitochondria. The synergistic action of these two mechanisms helps maintain cellular homeostasis and minimize the damage caused by oxidative stress. However, the specific mechanisms of interaction between Nrf2 and mitophagy, as well as the regulatory effects of HT, are not yet fully understood and require further investigation.

Human medical research is limited by the inability to conduct extensive experiments on the human body, making it difficult to obtain intestinal disease materials from humans. Therefore, finding suitable model animals has become a challenge. Piglets provide a structure and function like the human gut, making them an ideal choice in this field 21. By studying piglets to simulate human infantile gut diseases, scientists can gain a better understanding of the mechanisms and factors influencing the development of these diseases. Diquat (DQ) is a non-selective bipyridine herbicide that can induce oxidative stress response in animals. Currently, the DQ model has been widely used in studying the nutritional intervention effects on pig oxidative stress response 22. Therefore, this study aims to construct an intestinal oxidative stress model using DQ intraperitoneal injection and investigate the protective effect of HT on intestinal oxidative damage and its mechanism. The study will evaluate oxidative stress markers and intestinal barrier function to understand the impact of HT on intestinal oxidative stress. By examining the protective effect of HT, we hope to reveal its antioxidant mechanism and provide innovative ideas and strategies for the treatment of intestinal diseases. The findings of this study may serve as a valuable reference for clinical practice and fundamental research, deepen our understanding of the relationship between oxidative stress and intestinal diseases, and facilitate the development of novel treatment therapies.

## Materials and methods

### Chemicals and reagents

Hydroxytyrosol (HT) with a purity greater than 99% was obtained from Viablife Biotech Co (Hangzhou, China). DQ was purchased from Dr.Ehrenstorfer (Augsburg, Germany). Total antioxidant capacity (T-AOC), superoxide dismutase (SOD), glutathione peroxidase (GSH-Px), catalase (CAT), malondialdehyde (MDA), H_2_O_2,_ D-Lactic acid, and diamine oxidase (DAO) assay kits were purchased from Jiancheng Biochemical (Nanjing, China). The CCK-8 kit, ROS Assay Kit, H&E staining kits, and RIPA lysis buffer were purchased from Solarbio Company (Beijing, China). DMEM/F12, fetal bovine serum (FBS), penicillin-streptomycin (PS), Insulin-Transferrin-Selenium (ITS), epidermal growth factor (EGF), and PBS were purchased from Gibco (Maryland, USA). Antibodies against ZO-1, Occludin, Claudin1, p-Akt, and LC3 were obtained from Bioss (Beijing, China). Antibodies against HO-1, p62, ATG5, and ATG7 were obtained from Proteintech (Chicago, USA). Antibodies against Keap1, Nrf2, p-Nrf2, NQO1, PI3K, p-PI3K, Akt, PINK1, and Beclin1 were obtained from Beyotime (Shanghai, China). Antibodies against Parkin, β-actin, and secondary antibodies were obtained from Sangon Biotech (Shanghai, China). Alexa Fluor 488 dye-conjugated secondary antibody, Hoechst 33258 staining kit, LY294002, and Mdivi-1 were obtained from Beyotime (Shanghai, China). ML385 was purchased from AbMole (Houston, USA). SDS-PAGE gel and ECL kit were purchased from YangGuangBio (Beijing, China).

### Experimental animal and treatment

**Figure 1:**
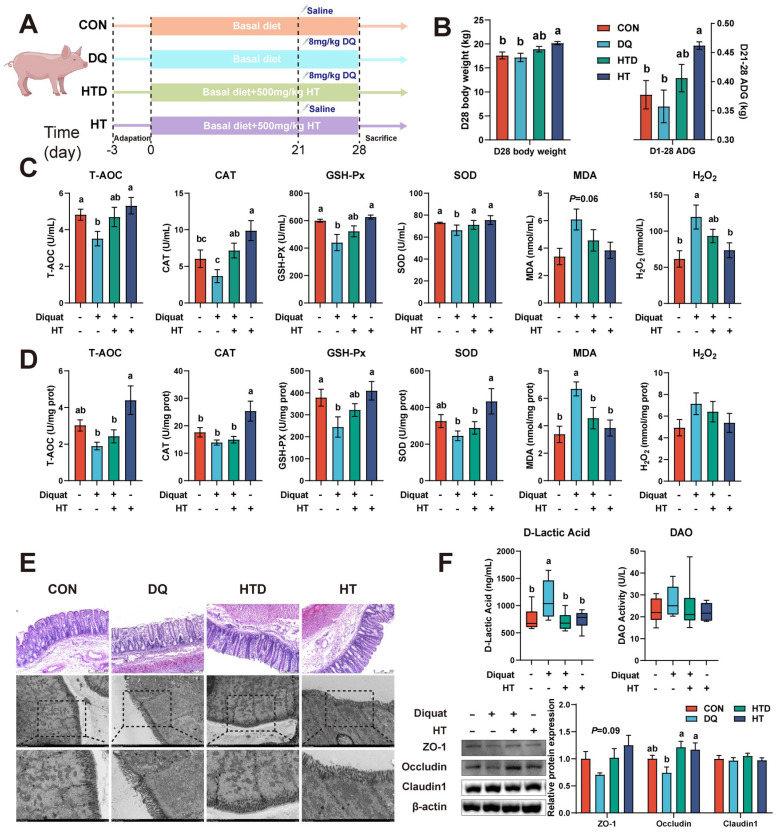
Hydroxytyrosol (HT) increased antioxidant capacity and alleviated intestinal oxidative damage in a piglet model. CON group: pigs receiving a basal diet and injected normal saline; DQ group: pigs receiving a basal diet and injected diquat (DQ); HTD group: pigs receiving a basal diet supplemented with 500 mg/kg HT and injected DQ; HT group: pigs receiving a basal diet supplemented with 500 mg/kg HT and injected normal saline. (A) The schematic diagram illustrates drug administration and experimental design in piglets. (B) Growth performance of piglets. ADG, average daily gain. (C) The level of total antioxidant capacity (T-AOC), catalase (CAT), glutathione peroxidase (GSH-Px), superoxide dismutase (SOD), malondialdehyde (MDA), and H_2_O_2_ in serum of piglets were determined by biochemical assay kits. (D) The level of T-AOC, CAT, GSH-Px, SOD, MDA, and H_2_O_2_ in the colonic mucosa of piglets was determined by ELISA. (E) Representative pictures of HE staining (200 × magnification) and TEM (2,000 and 4,000 × magnification) of colon tissue. (F) The abundance of intestinal barrier indicators includes D-lactic acid and diamine oxidase (DAO) measured by biochemical assay kits in serum, and tight junction proteins (ZO-1, Occludin, and Claudin-1) were measured by western blotting in the intestinal mucosa. Values are means ± SE. Different letters represent significant differences (*P* < 0.05).

All animal procedures were approved by the Animal Ethics Committee of the Institute of Animal Science, the Chinese Academy of Agricultural Sciences (IAS2021-228). Twenty-four weaned piglets (21-day old; 7.66 ± 0.85 kg; Duroc × Landrace × Yorkshire; male) were randomly allocated into 4 groups (Fig. 1A): (1) control group (CON), (2) DQ group (DQ), (3) HT group (HT) and (4) HT+DQ group (HTD). The CON and DQ groups were provided with a basal diet, whereas the HT and HTD group was given a basal diet supplemented with 500 mg/kg HT according to the pre-experiment. The basal diet met the NRC (2012) nutrient requirements for piglets. The DQ and HTD group received intraperitoneal injections of DQ (8 mg/kg body weight) on the 21st day, whereas the CON and HT group received normal saline in an equal volume. All piglets were housed in a clean and comfortable environment with *ad libitum* access to water and corresponding feed throughout the entire experiment. On the 28th day, all piglets were anesthetized and bled through the neck, and samples of colonic issues, mucosa, and chyme were collected.

### Cell culture and treatment

The intestinal porcine epithelial cell (IPEC-J2) was a generous gift from Dr. Zhengqun Liu (Tianjin Academy of Agriculture Sciences). IPEC-J2 cells were cultured in DMEM/F12 supplemented with 5% FBS, 5% PS, 1% ITS, and 5 ng/mL EGF in a 5% CO_2_ atmosphere at 37°C. Cells were treated with PBS or different pathway inhibitors (5 μM ML385, 10 μM LY294002, 1 μM Mdivi-1) for 1 h, and then treated with HT (50 μM) for 9 h, followed by DQ (75 μM) for 6 h. Then, cells were collected for ELISA, western blot, and transmission electron microscopy (TEM) after washed twice by cold PBS.

### Histological analysis

The colonic segments fixed in 4% paraformaldehyde were used to determine morphology using the H&E staining kit. Following the process of dehydration, embedding, sectioning, and staining, the colonic sections were examined using a Leica microscope. Additionally, the colonic segments and IPEC-J2 cells were fixed in a 2.5% glutaraldehyde solution and processed for TEM analysis. Ultrathin sections were made by professionals from Wuhan Servicebio Technology Co., Ltd (Wuhan, China).

### Cell viability assay

Cell viability was determined using a CCK-8 kit (Solarbio, Beijing China). IPEC-J2 cells (1 × 10^4^ cells/well) were cultured in 96-well plates. After 24 h, cells were treated with indicated agents for the indicated time. Then changed culture medium, 10 μL CCK-8 to each well, and incubated for 1.5 h. The absorbance at 450 nm was determined using a microplate reader (SpectraMax M2).

### Antioxidants and biochemical indexes

According to manufacturer guidelines, the antioxidants including T-AOC, SOD, GSH-Px, CAT, MDA, and H_2_O_2_ in serum, colonic mucosa, and IPEC-J2 cells, and permeability indicators including D-Lactic acid, DAO in serum were measured using biochemical assay kits (Jiancheng Biochemical, Nanjing, China).

### 16S rRNA amplicon sequencing for microbiome

Total genomic DNA was extracted from colonic chyme using MagPure Soil DNA LQ Kit (Magan) following the manufacturer's instructions. The extracted DNA was used as a template for PCR amplification of bacterial 16S rRNA genes with the barcoded primers and Takara Ex Taq (Takara). For bacterial diversity analysis, V3-V4 variable regions of 16S rRNA genes were amplified with universal primers 343F (5'-TACGGRAGGCAGCAG-3') and 798R (5'-AGGGTATCTAATCCT-3') for V3-V4 regions. Sequencing was performed on an Illumina NovaSeq 6000 with 250 bp paired-end reads and data processing was conducted by OE Biotech Co., Ltd. (Shanghai, China). QIIME2 software was used for alpha and beta diversity analysis. The unweighted Unifrac Principal coordinates analysis (PCoA) was used to estimate the beta diversity. Then Kruskal-Wallis statistical test was used to analyze the significant differences between different groups. The marker bacteria in each group were tested using the linear discriminant analysis effect size (LDA Effect Size, LEfSe), and the threshold of the LDA score was 4.0. The raw read data was submitted to the NCBI Sequence Read Archive database (PRJNA960710).

### Quantification of short-chain fatty acids (SCFAs)

Accurately weigh 1 g colonic chyme into a 10 mL centrifuge tube, and add 5 mL of ultrapure water. Shake vigorously for 30 min, then incubate overnight at 4°C. Centrifuge at 10,000 rpm for 10 min and transfer the supernatant to a new 10 mL centrifuge tube. Add 4 mL of ultrapure water to the precipitate and shake for another 30 min. Centrifuge at 10,000 rpm to collect the supernatant, then combine it with the previously collected supernatant in a 10 mL centrifuge tube for volumetric measurements. Transfer the supernatant, add in a 9:1 (900 uL supernatant + 100 uL 25% metaphosphoric acid) ratio into a 2 mL centrifuge tube, and let it react at room temperature for 3-4 h. After centrifugation, the supernatant was filtered through a 0.45-µm filter and SCFAs were quantified using Agilent 7890N GC. The calibration curve method was used for the quantitative determination of colonic SCFAs.

### ROS Measurement

After IPEC-J2 cells were treated with the indicated agent or ROS positive control (Rosup), the cell culture medium was removed and replaced with a serum-free culture medium containing 10 μM ROS fluorescent probe DCFH-DA. The cells are then incubated in a cell incubator at 37°C for 20 min. After incubation, the cells were washed 3 times with a serum-free cell culture medium. Subsequently, images were taken using an inverted fluorescence microscope (Leica DMi3000B), or the cells were collected and analyzed using a fluorescence microplate reader (SpectraMax M2, excitation wavelength: 488nm, emission wavelength: 525nm).

### Cell immunofluorescence

IPEC-J2 cells were seeded into chamber slides and treated with indicated agents. The cells were washed with PBS 3 times and fixed with 4% paraformaldehyde for 15 min, washed 3 times, and permeabilized with TBST for 30 min. After blocked with 10% goat serum for 30 min, the cells were incubated with primary antibody (Claudin1, 1:100) overnight at 4 °C. The cells were then incubated with Alexa Fluor 488 dye-conjugated secondary antibody (1:150) at 37°C for 1 h away from light. Washed 3 times with PBS, the cells were then counterstained with Hoechst 33258 to stain the nuclei. IPEC-J2 cells were subsequently examined with an inverted fluorescence microscope (Leica DMi3000B).

### Western blotting

Total proteins were extracted from colonic mucosa and IPEC-J2 using RIPA lysis buffer. The total protein was separated using SDS-PAGE gel and transferred to 0.45 μm PVDF membranes (Millipore, USA). Blocked with 5% skim milk for 2 h, the membranes were incubated with primary antibodies (ZO-1, Occludin, Claudin1, β-actin, Keap1, Nrf2, p-Nrf2, HO-1, NQO1, Akt, p-Akt, PI3K, p-PI3K, PINK1, Parkin, Beclin1, p62, LC3, ATG5, ATG7) overnight at 4°C. The next day, the membranes were incubated with a secondary antibody at room temperature for 1 h. Finally, protein bands were detected using an ECL kit, and band density was quantified using ImageJ software.

### Statistical analysis

Data are presented as means ± SE. Student's t-test was used for comparisons between two groups, while one-way analysis of variance analysis (ANOVA) with the Tukey test was used for comparisons between multiple groups. All statistical significance was set at *P* < 0.05.

## Results

### HT treatment protects the gut barrier from damage caused by DQ-induced oxidative stress by enhancing antioxidant capacity

We initially induced oxidative stress through intraperitoneal injection of DQ, leading to a reduction in serum antioxidant capacity. Specifically, the levels of T-AOC, SOD, and GSH-Px decreased, while the levels of MDA and H_2_O_2_ increased, indicating the successful establishment of the DQ-induced oxidative stress model (Fig. 1C). The HT treatment led to elevated levels of T-AOC, SOD, and CAT, accompanied by reduced levels of MDA and H_2_O_2_ in the serum (Fig. 1C), demonstrating the HT intervention effectively ameliorated the decline in antioxidant capacity caused by DQ. The similarly protective effect of HT supplementation on colon tissue against DQ-induced oxidative stress was also observed, manifesting as increased levels of GSH-Px levels, and decreased content of MDA (Fig. 1D). Besides, compared to the DQ group, the addition of HT alone significantly improved the D28 body weight and average daily gain (ADG; Fig. 1B).

Next, H&E staining and TEM were conducted to assess the pathological alterations in the colon. As illustrated in Fig. 1E, the DQ group exhibited notable damage and disruption to the colonic mucosa and surface epithelium compared to the control group. The ultrastructure appeared to be disrupted and damaged in the DQ group, characterized by shortened or even destroyed microvilli, deformation of some cellular structures, and swollen mitochondria. However, the HT treatment prevented the deterioration of intestinal pathological morphology caused by DQ damage, resulting in well-preserved ultrastructure (Fig. 1E). Moreover, we found that HT supplementation reversed the high level of D-lactic acid, an indicator of gut permeability 23, induced by DQ injection (Fig. 1F). This result, in conjunction with Western blotting data demonstrating increased expressions of colonic ZO-1 and Occludin after HT administration following DQ injection (Fig. 1F), suggests that HT treatment enhances the integrity of gut barrier and even rescues DQ-induced damage to the gut barrier. These findings indicate that HT can improve the antioxidant capacity and reverse DQ-induced damage to the gut barrier.

### The impact of HT treatment or DQ injection on gut microbe composition is limited

**Figure 2:**
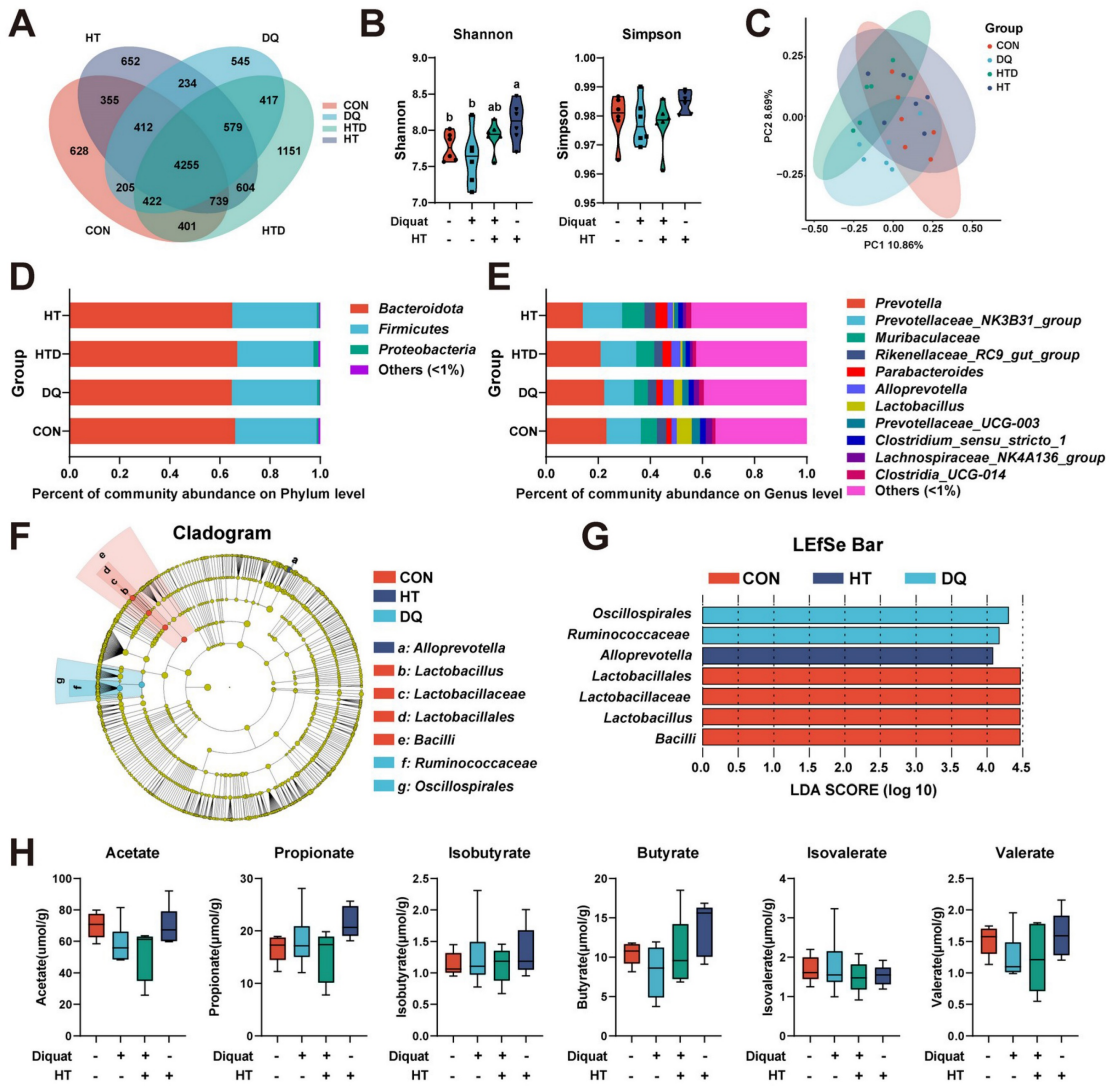
Hydroxytyrosol (HT) had little effect on intestinal microbiota and short-chain fatty acids (SCFAs) in a piglet model. CON group: pigs receiving a basal diet and injected normal saline; DQ group: pigs receiving a basal diet and injected diquat (DQ); HTD group: pigs receiving a basal diet supplemented with 500 mg/kg HT and injected DQ; HT group: pigs receiving a basal diet supplemented with 500 mg/kg HT and injected normal saline. (A) Venn diagram of OTU distribution. (B) The alpha diversity indices (Shannon and Simpson) of intestinal microbiota. (C) The beta diversity using the unweighted Unifrac Principal coordinates analysis (PCoA). (D) Microbiota composition at the phylum level. (E) Microbiota composition at the genus level. (F) Cladogram and (G) LDA distribution. (H) The abundance of SCFAs was measured with gas chromatography (GC). Values are means ± SE. Different letters represent significant differences (*P* < 0.05).

Given the critical role of microbiota in maintaining host health and serving as intermediaries between the host and dietary factors 24, we concentrated on the composition of microbiota and microbiota-derived short-chain fatty acids (SCFAs). The OTU Venn diagram and analyses of alpha and beta diversities demonstrated that neither DQ stimulation nor HT treatment significantly impacted microbial diversity, except for the group treated solely with HT, which exhibited a higher Shannon index compared to the CON group (Fig. 2A-C). At the phylum level, *Bacteroidota*, *Firmicutes*, and *Proteobacteria* collectively comprised approximately 99.4% of the total colonic bacterial community (Fig. 2D). At the genus level, *Prevotella*, *Prevotellaceae_NK3B31_group*, *Muribaculaceae*, *Rikenellaceae_RC9_gut_group*, and *Parabacteroides* emerged as the predominant genera (Fig. 2E). However, there were no significant differences observed in microbial abundance at phylum and genus levels. Given this, we further investigated the composition of the microbiota using LEfSe (LDA score threshold: 4.0) to identify marker bacteria that could potentially contribute to the observed effects. We observed enrichment of *Lactobacillales*, *Lactobacillaceae*, *Lactobacillus*, and *Bacilli* in the CON group, *Oscillospirales* and *Ruminococcaceae* in the DQ group, and *Alloprevotella* in the HT group. (Fig. 2F-G). These findings suggest that specific bacteria may be more abundant in certain groups and could play a role in the observed differences in intestinal health. Furthermore, targeted metabolomics analysis showed no significant changes in the levels of SCFAs among the treatment groups (Fig. 2H). This lack of change in SCFAs levels suggests the protective effects of HT on DQ-induced intestinal oxidative damage may not be primarily mediated through alterations in SCFAs metabolism. Considering the rather subtle changes in the gut microbiota and the absence of significant alterations in SCFAs levels, we redirected our focus from microbial mediation towards elucidating the potential mechanism through which HT administration directly mitigates DQ-induced oxidative stress damage in the intestine.

### HT alleviates intestinal oxidative damage in IPEC-J2 cells

We employed an in vitro DQ-induced oxidative stress model of porcine intestinal epithelial cells to confirm the direct protective effect of HT administration. Firstly, we evaluated the time and concentration parameters for inducing oxidative stress in IPEC-J2 cells using DQ. As depicted in Fig. S1A, following treatment with DQ for 6 h, a notable decline in cell viability was observed, particularly evident when the concentration of DQ surpassed 75 μM. To explore the potential protective effects of varying concentrations and durations of HT administration against DQ-induced cell damage, we pre-incubated the cells with five different concentrations of HT for different periods. Subsequently, the IPEC-J2 cells were then treated with 75 μM DQ for 6 h following the pre-incubation. The results showed that pre-incubation with a minimum concentration of 50 μM HT for at least 9 h was necessary to achieve beneficial effects from HT administration (Fig. S1B).

**Figure 3:**
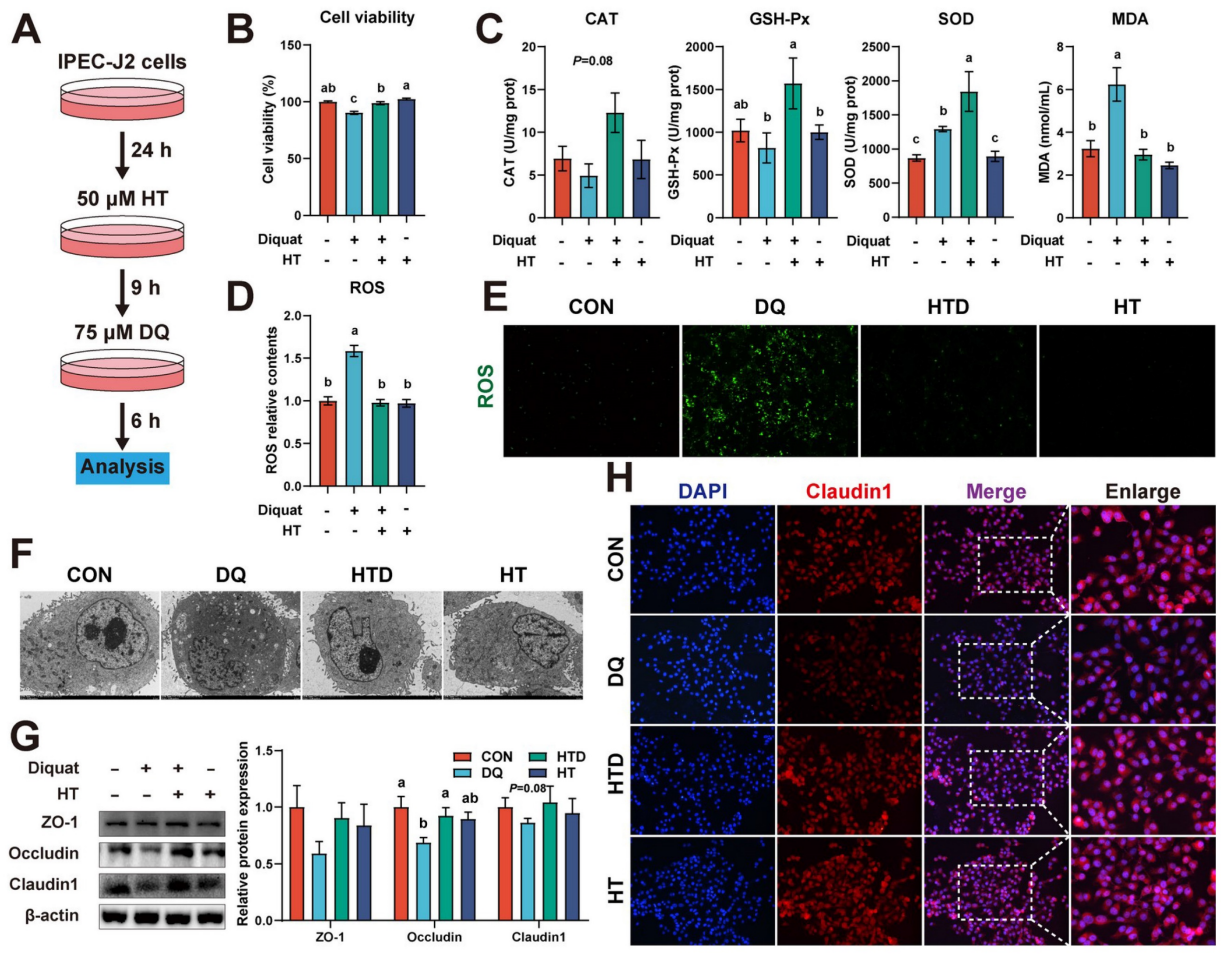
Hydroxytyrosol (HT) increased antioxidant capacity and alleviated oxidative stress in intestinal epithelial cells (IPEC-J2). (A) Schematic diagram illustrates drug administration in IPEC-J2 cells (B) The cell viability of IPEC-J2 cells was determined by CCK-8 assay. (C) The levels of catalase (CAT), glutathione peroxidase (GSH-Px), superoxide dismutase (SOD), and malondialdehyde (MDA) were determined by biochemical assay kits. (D) The relative content and (E) staining of cellular reactive oxygen species (ROS) was determined by ROS Assay Kit. (F) Cellular ultrastructure was visualized using TEM (2,000 × magnification). (G) Western blotting determined the protein expression and quantitation of ZO-1, Occludin, and Claudin-1. (H) Representative immunofluorescence images of Claudin-1. Values are means ± SE. Different letters represent significant differences (*P* < 0.05).

Next, we selected a concentration of 50 μM HT and a treatment duration of 9 h for the subsequent experiments (Fig. 3A). The protective effect of HT treatment on cell viability impaired by DQ was reaffirmed (Fig. 3B). Furthermore, DQ treatment led to elevated levels of SOD and MDA, whereas HT treatment resulted in increased levels of GSH-Px and SOD (Fig. 3C). DQ treatment also strongly stimulated ROS production, but this effect was completely abolished by pretreatment with HT (Fig. 3D). Further confirmation of this outcome was obtained through fluorescence microscopy, revealing a reduction in the fluorescence intensity of ROS after HT pretreatment (Fig. 3E). TEM examination unveiled that DQ caused damage to intracellular organelles and the formation of numerous vesicles, whereas HT maintained cell integrity without any signs of damage (Fig. 3F). Moreover, western blotting demonstrated that DQ treatment resulted in a decrease in the abundance of Occludin and Claudin1, which was reversed by HT treatment (Fig. 3G). A similar trend was observed in immunofluorescence staining for Claudin1 (Fig. 3H). These data suggested that HT can enhance the antioxidant capacity and directly alleviate oxidative damage in the intestinal epithelium.

### HT alleviates intestinal oxidative damage by activating the Nrf2 pathway

**Figure 4:**
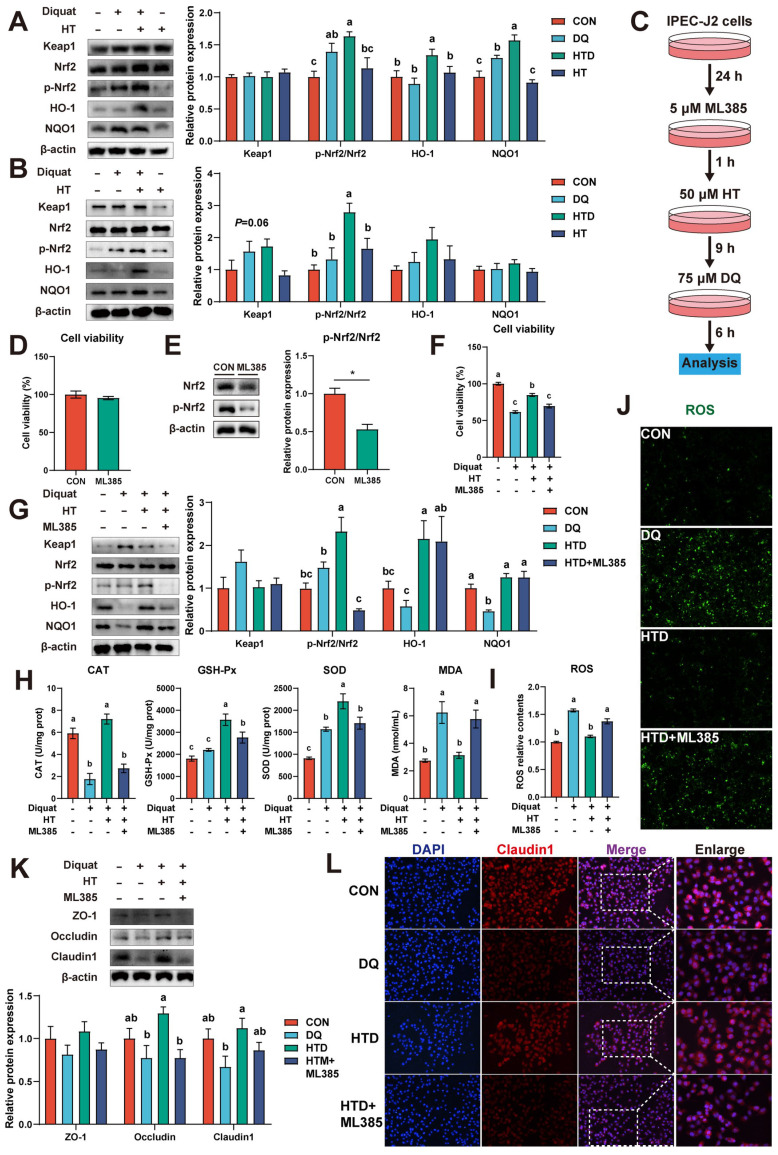
Hydroxytyrosol (HT) increased antioxidant capacity and alleviated oxidative stress through the Nrf2 signaling pathway. (A) Protein expression and quantitation of Keap1, Nrf2, p-Nrf2, HO-1, and NQO1 were determined by western blotting in the intestinal mucosa of piglets. (B) Protein expression and quantitation of Keap1, Nrf2, p-Nrf2, HO-1, and NQO1 were determined by western blotting in IPEC-J2 cells. (C) The schematic diagram illustrates drug administration in IPEC-J2 cells. ML385, Nrf2 pathway inhibitor. (D) The cell viability of IPEC-J2 cells treated with ML385 was determined by CCK-8 assay. (E) Protein expression and quantitation of Nrf2 and p-Nrf2 in IPEC-J2 cells treated with ML385 were determined by western blotting. (F) The cell viability of IPEC-J2 cells was determined by CCK-8 assay in the Nrf2 pathway inhibition experiment. (G) Protein expression and quantitation of Keap1, Nrf2, p-Nrf2, HO-1, and NQO1 were determined by western blotting in the Nrf2 pathway inhibition experiment. (H) The levels of catalase (CAT), glutathione peroxidase (GSH-Px), superoxide dismutase (SOD), and malondialdehyde (MDA) were determined by biochemical assay kits. (I) The relative content and (J) staining of cellular reactive oxygen species (ROS) were determined by ROS Assay Kit. (K) Western blotting determined the protein expression and quantitation of ZO-1, Occludin, and Claudin-1. (L) Representative immunofluorescence images of Claudin-1. Values are means ± SE. Different letters and * represent significant differences (*P* < 0.05).

The Nrf2 signaling pathway, the most important and classic signaling pathway, plays a crucial role in regulating the level of ROS 25. We examined the expression of Nrf2 and its downstream proteins in piglets. Results showed that DQ treatment resulted in upregulation of p-Nrf2 and NQO1 protein expression in the colon compared to the CON group in piglets, and their expression levels further increased after HT treatment. Additionally, HT treatment increased the expression of HO-1 (Fig. 4A). In IPEC-J2 cells, HT treatment also increased the protein levels of p-Nrf2 (Fig. 4B). These results suggest that HT treatment can activate the Nrf2 pathway in the progress of improving intestinal oxidative damage. To further confirm the roles of the Nrf2 pathway in the antioxidant damage process of HT, we performed experiments to investigate whether pathway inhibitors could inhibit the antioxidant effect of HT in IPEC-J2 cells.

Furthermore, the Nrf2 pathway inhibitor ML385 was employed to confirm the roles of the Nrf2 pathway in the antioxidant damage process of HT (Fig. 4C). Data demonstrated that the inhibitor ML385 alone did not significantly affect cell viability but significantly inhibited the protein expression of p-Nrf2 (Fig. 4D-E). Compared with the DQ group, HT treatment increased the cell viability of IPEC-J2 cells, consistent with the results in Fig. 3B. However, ML385 eliminated the enhancement of cell viability caused by HT treatment (Fig. 4F). Although this study found that inhibiting Nrf2 expression had no significant effect on HO-1 and NQO1 (Fig. 4G), it significantly suppressed the enhanced antioxidant capacity of HT, such as reducing CAT, GSH-Px and SOD activity, and increasing MDA content (Fig. 4H). Simultaneously, the decrease of ROS production observed in Fig. 3D by HT treatment was inhibited by ML385, which was consistent with the fluorescence microscopy of ROS (Fig. 4I-J).

Additionally, we found that HT treatment effectively prevented the decline in protein expression of Occludin and Claudin1 in IPEC-J2 cells, and ML385 counteracted these protective effects (Fig. 4K). This was confirmed by the immunofluorescence staining of Claudin1 (Fig. 4L). Taken together, these results indicate that HT improves intestinal oxidative damage by activating the Nrf2 signaling pathway.

### Nrf2 signaling-mediated HT attenuates intestinal oxidative damage depending on the activation of the PI3K/Akt pathway

**Figure 5:**
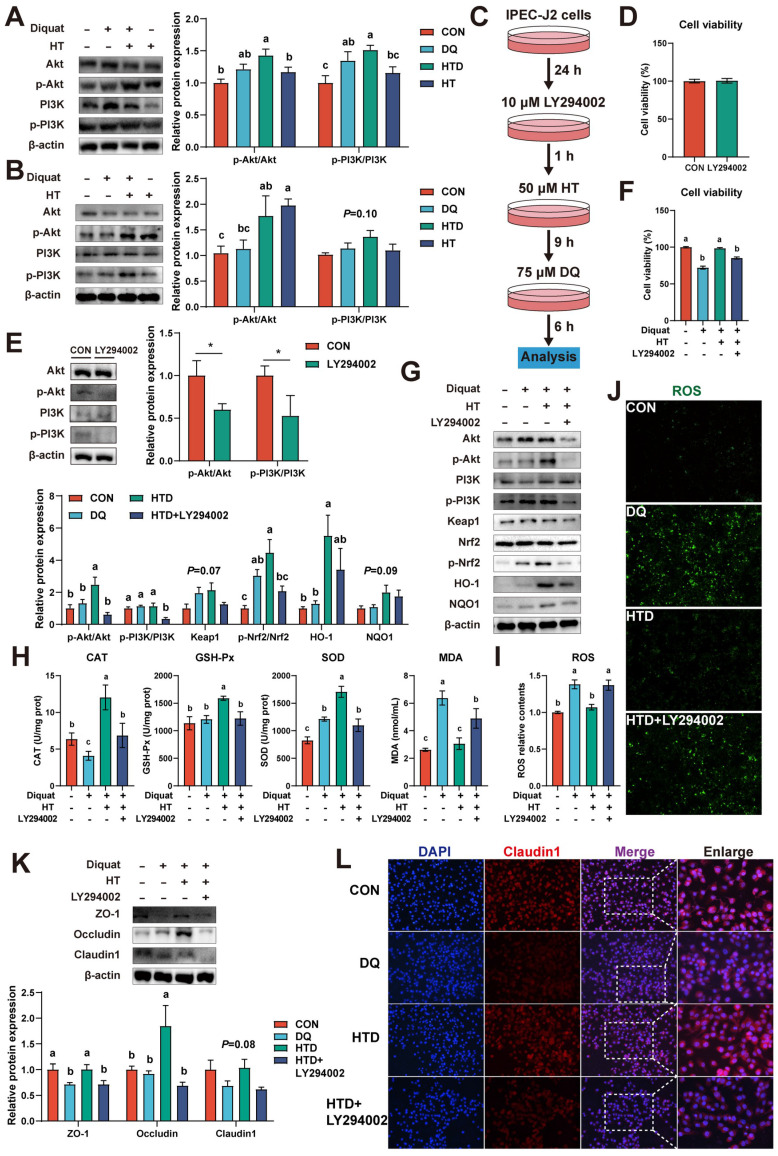
Hydroxytyrosol (HT) increased antioxidant capacity and alleviated oxidative stress through the PI3K/Akt-Nrf2 signaling pathway. (A) Protein expression and quantitation of Akt, p-Akt, PI3K, and p-PI3K were determined by western blotting in the intestinal mucosa of piglets. (B) Protein expression and quantitation of Akt, p-Akt, PI3K, and p-PI3K were determined by western blotting in IPEC-J2 cells. (C) The schematic diagram illustrates drug administration in IPEC-J2 cells. LY294002, PI3K/Akt pathway inhibitor. (D) The cell viability of IPEC-J2 cells treated with LY294002 was determined by CCK-8 assay. (E) Protein expression and quantitation of Akt, p-Akt, PI3K, and p-PI3K in IPEC-J2 cells treated with LY294002 were determined by western blotting. (F) The cell viability of IPEC-J2 cells was determined by CCK-8 assay in the PI3K/Akt pathway inhibition experiment. (G) Protein expression and quantitation of Akt, p-Akt, PI3K, p-PI3K, Keap1, Nrf2, p-Nrf2, HO-1, and NQO1 were determined by western blotting in PI3K/Akt pathway inhibition experiment. (H) The levels of catalase (CAT), glutathione peroxidase (GSH-Px), superoxide dismutase (SOD), and malondialdehyde (MDA) were determined by biochemical assay kits. (I) The relative content and (J) staining of cellular reactive oxygen species (ROS) were determined by ROS Assay Kit. (K) Western blotting determined the protein expression and quantitation of ZO-1, Occludin, and Claudin-1. (L) Representative immunofluorescence images of Claudin-1. Values are means ± SE. Different letters and * represent significant differences (*P* < 0.05).

Evidence has been documented indicating that polyphenols modulate the Nrf2 signaling pathway, thereby exerting antioxidant effects, contingent upon the activation of the PI3K/Akt pathway 26, 27. Hence, we investigated the expression of p-PI3K, PI3K, p-Akt, and Akt in both piglets and cellular experiments. In the piglet experiment, it was found that DQ and HT treatments led to elevated protein levels of p-PI3K and p-Akt (Fig. 5A). Similarly, in IPEC-J2 cells, HT treatment significantly increased the protein levels of p-Akt (Fig. 5B).

To further validate the involvement of the PI3K/Akt pathway in the antioxidant effects of HT, we employed the PI3K/Akt pathway inhibitor LY294002 (Fig. 5C). The inhibitor LY294002 alone had no significant effect on cell viability, but significantly decreased the protein levels of p-PI3K and p-Akt (Fig. 5D-E). As anticipated, upon inhibition of the PI3K/Akt pathway, both the phosphorylation of Nrf2 and the expression of downstream pathway proteins notably decreased, consequently inhibiting the beneficial effect of HT on cell viability (Fig. 5F-G). Moreover, antioxidant capacity experienced a significant decline following LY294002 treatment, as evidenced by reduced levels of CAT, GSH-Px, and SOD, and increased MDA levels (Fig. 5H). Consistently with these results, LY294002 treatment resulted in a substantial increase in ROS levels, as confirmed by fluorescence detection (Fig. 5I).

In addition, the expression of tight junction proteins (ZO-1, Occludin, Claudin1) was significantly reduced by LY294002 administration, consequently inhibiting the improvement effect of HT, as confirmed by the disappearance of the improvement in Claudin1 immunofluorescence (Fig. 5K-L). Overall, these results indicate that HT exerts its antioxidant function by activating the PI3K/Akt pathway to modulate the Nrf2 pathway.

### HT alleviates intestinal oxidative damage through mitophagy

**Figure 6:**
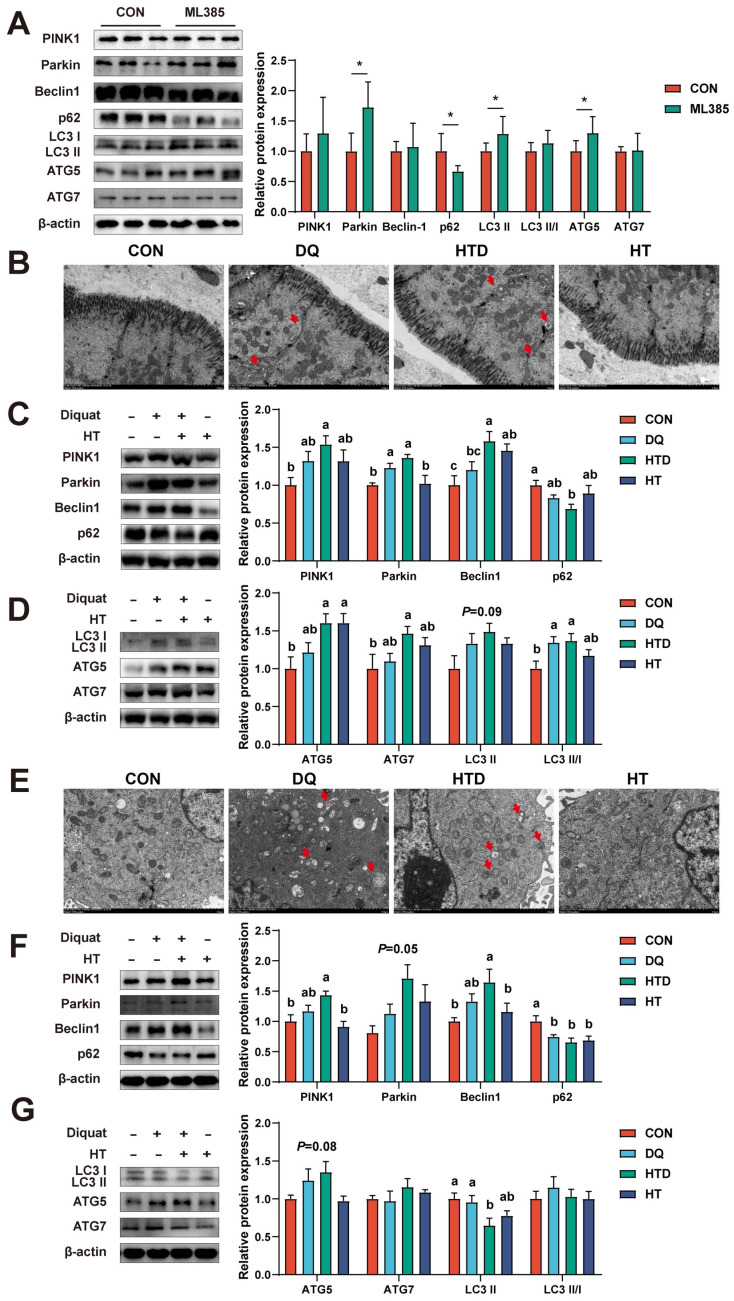
Hydroxytyrosol (HT) activated mitophagy to regulate oxidative stress in piglets and IPEC-J2 cells. (A) Protein expression and quantitation of PINK1, Parkin, Beclin1, p62, LC3 I/II, ATG5, and ATG7 were determined by western blotting in IPEC-J2 cells treated with Nrf2 pathway inhibitor ML385. (B) Cellular ultrastructure in the piglet's colon was visualized using TEM (4,000 × magnification). Red arrows indicate mitophagosomes. (C-D) Protein expression and quantitation of PINK1, Parkin, Beclin1, p62, LC3 I/II, ATG5, and ATG7 were determined by western blotting in the intestinal mucosa of piglets. (E) Cellular ultrastructure in IPEC-J2 cells was visualized using TEM (4,000 × magnification). Red arrows indicate mitophagosomes. (F-G) Protein expression and quantitation of PINK1, Parkin, Beclin1, p62, LC3 I/II, ATG5, and ATG7 were determined by western blotting in IPEC-J2 cells. Values are means ± SE. Different letters and * represent significant differences (*P* < 0.05).

Mitophagy is an essential cellular metabolic process that contributes to the maintenance of intracellular homeostasis and protects cells from oxidative stress damage by eliminating damaged mitochondria 28. In studying the Nrf2 signaling pathway, we have discovered that inhibition of the Nrf2 antioxidant pathway could induce mitophagy. Specifically, cell experiments have shown that the Nrf2 pathway inhibitor ML385 significantly increased the levels of mitophagy-related proteins, such as Parkin, LC3 II, and ATG5, while reducing the expression of the autophagic substrate p62 (Fig. 6A). To further investigate this finding, we conducted tests on mitophagy in piglets and cell experiments. TEM analysis revealed that DQ and HT treatments induced the formation of mitophagosomes in the colon of piglets (Fig. 6B). Meanwhile, DQ treatment increased the expression of mitophagy-related proteins, such as Parkin and LC3 II/I, and HT treatment further elevated the expression levels of these proteins (Fig. 6C-D). Similarly, we also observed an increase in mitophagosome numbers in IPEC-J2 cells after DQ and HT treatment, but more in the HTD group (Fig. 6E). DQ treatment also increased the expression of mitophagy-related proteins, and the expression of PINK1, Parkin, and Beclin1 was significantly increased after HT treatment (Fig. 6F-G). These findings suggested a close relationship between the Nrf2 pathway and mitophagy in the antioxidant process, and mitophagy participates in the HT-mediated antioxidant process.

**Figure 7:**
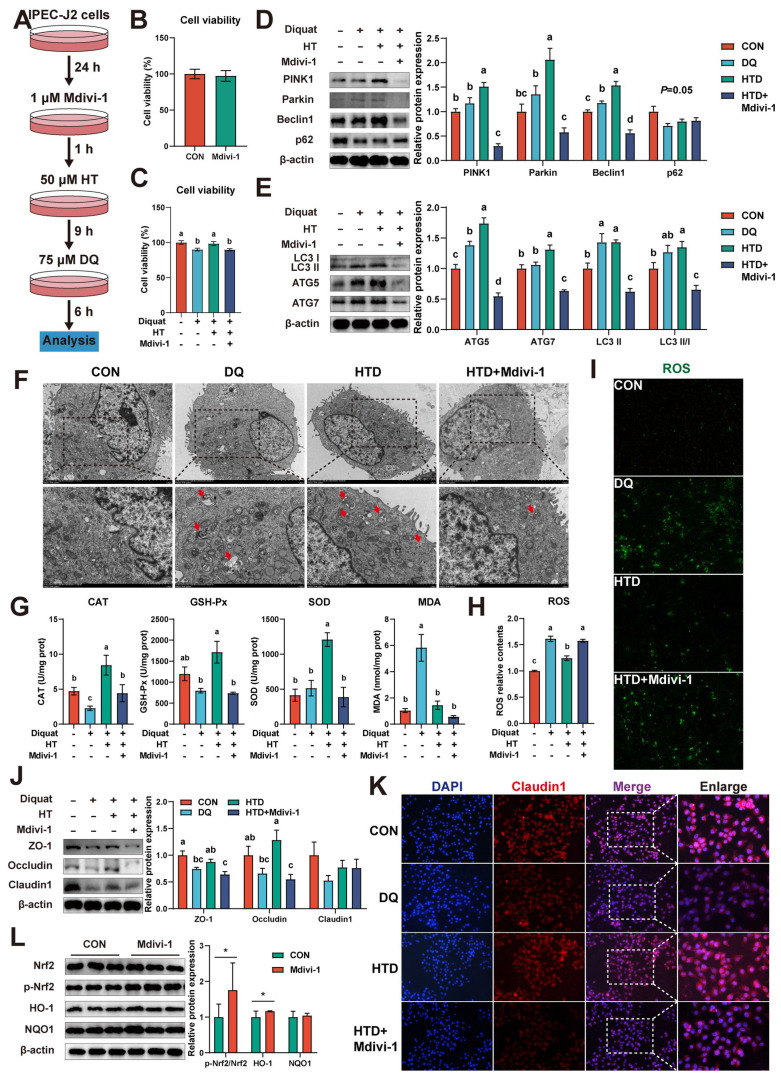
Hydroxytyrosol (HT) increased antioxidant capacity and alleviated oxidative stress by regulating mitophagy. (A) The schematic diagram illustrates drug administration in IPEC-J2 cells. Mdivi-1, mitophagy inhibitor. (B) The cell viability of IPEC-J2 cells treated with Mdivi-1 was determined by CCK-8 assay. (C) The cell viability of IPEC-J2 cells was determined by CCK-8 assay in the mitophagy inhibition experiment. (D-E) Protein expression and quantitation of PINK1, Parkin, Beclin1, p62, LC3 I/II, ATG5, and ATG7 were determined by western blotting in IPEC-J2 cells. (F) Cellular ultrastructure in IPEC-J2 cells was visualized using TEM (2,000 and 4,000 × magnification). Red arrows indicate mitophagosomes. (G) The levels of catalase (CAT), glutathione peroxidase (GSH-Px), superoxide dismutase (SOD), and malondialdehyde (MDA) were determined by biochemical assay kits. (H) The relative content and (I) staining of cellular reactive oxygen species (ROS) were determined by the ROS Assay Kit. (J) Western blotting determined the protein expression and quantitation of ZO-1, Occludin, and Claudin-1. (K) Representative immunofluorescence images of Claudin-1. (L) Protein expression and quantitation of Nrf2, p-Nrf2, HO-1, and NQO1 were determined by western blotting in IPEC-J2 cells treated with Mdivi-1. Values are means ± SE. Different letters and * represent significant differences (*P* < 0.05).

To further determine the role of mitophagy in the HT-mediated improvement of oxidative stress, we first pre-treated IPEC-J2 cells with 1 μM mitophagy inhibitor Mdivi-1 and then performed subsequent experiments (Fig. 7A). It should be noted that the addition of Mdivi-1 alone had no significant effect on cell viability (Fig. 7B). Firstly, we found that the HT-mediated improvement of cell viability was abolished when mitophagy was inhibited, resulting in reduced cell viability (Fig. 7C). Western blotting analysis of mitophagy-associated proteins revealed that DQ treatment increased the levels of Beclin1, ATG5, and LC3 II, while HT treatment further enhanced the expression of these proteins and significantly increased the levels of PINK1, Parkin, ATG7, and LC3 II/I. However, after treatment with Mdivi-1, the expression of all these proteins was significantly decreased (Fig. 7D-E).

Correspondingly, TEM analysis showed that DQ treatment led to an increase in mitophagosome numbers, which further increased under HT treatment, whereas Mdivi-1 inhibited vesicle formation (Fig. 7F). Moreover, we examined the effect of Mdivi-1 on antioxidant capacity and found that Mdivi-1 blocked the enhanced antioxidant capacity of HT, such as significantly reducing the activities of CAT, GSH-Px, and SOD, and interestingly, the MDA content decreased simultaneously (Fig. 7G). Additionally, mitophagy inhibition resulted in an expected increase in ROS levels (Fig. 7H-I), because damaged mitochondria could not be cleared through autophagy, leading to the production of excessive ROS. Lastly, Mdivi-1 caused a decrease in tight junction protein expression, counteracting the enhanced tight junction protein expression induced by HT in DQ-treated cells, and cell immunofluorescence of Claudin1 also confirmed this (Fig. 7J-K). Taken together, the antioxidant potential of HT was suppressed upon mitophagy inhibition. These findings reveal the significant role of mitophagy in the alleviation of oxidative stress by HT.

Additionally, we noted upregulation of p-Nrf2 and HO-1 protein expression upon inhibition of mitophagy (Fig. 7L). Furthermore, when Nrf2 expression was repressed, mitophagy was enhanced. Both instances led to a decrease in antioxidant enzyme activity, an increase in ROS levels, and a reduction in tight junction protein expression, which collectively suppressed cellular activity. These data indicate that the collaborative regulation of the Nrf2 signaling pathway and mitophagy is indispensable to maintaining intracellular redox balance.

## Discussion

Oxidative stress appears to be involved in almost all intestinal diseases such as IBD and CRC. The occurrence, development, and prognosis of intestinal diseases are closely related to oxidative stress 29. This field of research is expected to become a new hotspot in future medical studies, bringing better treatment options for patients. Consequently, intervention strategies targeting the oxidative stress pathway, such as dietary or exogenous drug interventions, hold great potential for the treatment of intestinal diseases 30. Given the excellent properties of HT, it is expected to be a dominant drug for treating oxidative stress-related diseases. Piglets serve as an ideal model for simulating human intestinal diseases and have great potential and value in promoting medical research and clinical practice 31. Therefore, this study selected piglets as experimental subjects to explore the improvement of oxidative stress by HT and its potential mechanisms. We found that intraperitoneal injection of DQ could induce oxidative stress in piglets, leading to decreased antioxidant capacity and intestinal damage. However, the addition of HT could effectively prevent these adverse phenomena. To further reveal the mechanism of action of HT, we conducted experiments using *in vitro* intestinal epithelial cells IPEC-J2. The results showed that HT mainly improved oxidative damage by regulating the PI3K/Akt-Nrf2 signaling pathway and activating mitophagy. These findings provide new insights and strategies for the treatment of intestinal oxidative stress diseases, with significant scientific value and clinical significance.

Under normal conditions, ROS maintains a low concentration and serves as a signaling molecule 32. Under oxidative stress conditions, the amount of ROS rapidly increases. ROS causes oxidative damage to cellular molecules such as DNA, lipids, and proteins 33. Cells concurrently activate antioxidant defense systems to counteract oxidative damage, promoting target antioxidant gene expression, such as SOD, CAT, and GSH-Px. These antioxidant enzymes clear ROS within cells, maintaining redox homeostasis and thereby reducing cell damage caused by oxidative stress 34. Previous studies have shown that polyphenols, such as resveratrol, caffeic acid, and chlorogenic acid, can improve intestinal oxidative stress by increasing antioxidant enzyme activity 35-37. Like these polyphenols, HT is a potential antioxidant stress drug that has shown significant efficacy and safety in prospective clinical trials in recent years 38, 39. This study reveals the mechanism of HT in improving oxidative stress. It was found that compared with the DQ group, the HT treatment group had higher activities of antioxidant enzymes including SOD, CAT, and GSH-Px, and further inhibited the excessive production of ROS or MDA *in vitro* and* in vivo*. In addition, HT could enhance gut barrier function by increasing the expression of tight junction proteins (ZO-1, Occludin) and maintaining gut structure integrity, thereby improving the body weight of piglets. The gut barrier is the first line of defense, which can reduce the risk of pathogen invasion and the occurrence of gut diseases 40.

Similarly, other studies have also found that HT can increase antioxidant enzyme activity, reduce the content of oxidative products, and enhance gut barrier function 11, 12. These results indicate that HT has potential antioxidant stress function and helps maintain gut health. Although this study confirms that HT can improve oxidative stress, its mechanism of action is not yet fully understood. Microbiota and its metabolites are another important factor affected by oxidative stress, and they are also potential pathways for HT to improve oxidative stress 41. However, there were no significant differences in the microbiota and its metabolites among different groups, which may be related to the choice of animal models and interspecies response differences. Additionally, the small sample size and focus on abundant microbial populations in the study may have failed to capture subtle changes. Therefore, we redirected our focus from microbial mediation to investigating the potential direct mechanisms through which HT administration might mitigate DQ-induced intestinal oxidative damage in this study. We propose that HT may exert its protective effects through direct antioxidant properties, modulation of host cell signaling pathways, or other direct interactions with the intestinal tissue, which warrants further investigation.

Naturally, we have focused on the crucial antioxidant signaling pathway - the Nrf2 pathway, which plays a vital role in countering oxidative stress-related diseases, including IBD 42. In normal circumstances, Keap1 binds to Nrf2 and mediates its ubiquitination and degradation 43. When ROS levels increase due to oxidative stress, the interaction between Keap1 and Nrf2 is disrupted, leading to Nrf2 phosphorylation and entry into the cell nucleus. In the nucleus, Nrf2 binds to the antioxidant response element (ARE), thereby initiating the transcription of antioxidant genes, reducing ROS levels, and alleviating oxidative stress damage 44, 45. Our research findings demonstrated that DQ treatment can enhance the levels of p-Nrf2 and NQO1 in *vitro* and *in vivo*, but reduces the antioxidant enzyme activity, leading to persistently elevated ROS levels and subsequent damage to the intestinal barrier function. This suggests that although DQ activates the Nrf2 antioxidant pathway, it cannot completely offset the damage caused, or other regulatory factors may be influencing the activity of antioxidant enzymes, such as the possibility that elevated ROS levels induced by DQ treatment consumed a large number of antioxidant enzymes. Additionally, even though Nrf2 protein expression increased, there may be unknown reasons, such as changes in regulatory proteins or feedback regulation of enzyme activity, leading to the lack of a significant increase in antioxidant enzyme activity. It is noteworthy that HT treatment not only further elevates the expression of related proteins but also increases antioxidant enzyme activity, thereby maintaining the integrity of the intestinal barrier and enhancing the survival rate of intestinal cells. Therefore, drug intervention to further increase Nrf2 expression and activity may be an effective strategy for treating intestinal oxidative stress. In the IPEC-J2 oxidative stress model, we added the Nrf2 pathway inhibitor ML385, which significantly suppressed the upregulation of p-Nrf2 and antioxidant enzyme levels by HT and increased ROS levels. Moreover, the protective effects of HT against DQ-induced oxidative stress and intestinal cell damage were also substantially inhibited by ML385. These data thoroughly confirm that Nrf2 can enhance the elimination of ROS by increasing antioxidant enzymes downstream, playing an indispensable role in the improvement of intestinal oxidative stress disease with HT. In addition, the regulatory effect of HT on the Nrf2 pathway has also been found in other disease models, including Alzheimer's disease 46, bovine mastitis 47, and obesity 48. Hence, future studies can further explore the regulatory mechanism of the Nrf2 signaling pathway in intestinal oxidative stress to provide new strategies for the clinical treatment of related diseases.

The PI3K/Akt signaling pathway significantly contributes to the regulation of various pathophysiological processes such as metabolism, oxidative stress, and immune inflammation 49. Increasingly studies have shown that the PI3K/Akt-Nrf2 signaling pathway plays a dominant role in maintaining redox homeostasis by inhibiting ROS generation 50-53. Some polyphenols, such as chlorogenic acid, arbutin, hesperetin, and formononetin, have been shown to exert antioxidant effects through the PI3K/Akt-Nrf2 signaling pathway 52, 54-56. This suggests that polyphenolic compounds have potential antioxidant effects, which help maintain cellular stability. Therefore, we hypothesized that HT also exerts antioxidant effects through this pathway, as we have confirmed that Nrf2 is involved in this process. Firstly, we found that HT treatment could alleviate oxidative stress-induced cell damage both *in vitro* and *in vivo* while increasing the abundance of phosphorylated PI3K and Akt. This indicates that HT treatment activated the PI3K/Akt signaling pathway and may be involved in the activation of the Nrf2 antioxidant pathway. In a previous study, HT was also reported to induce antioxidant enzymes and Nrf2 translocation via PI3K/Akt pathways in HepG2 cells 57. Next, to further confirm the pathway through which HT induces Nrf2 activation, we performed an inhibitor experiment using the IPEC-J2 oxidative stress model. The results showed that the activation of Nrf2 in IPEC-J2 cells by HT was inhibited by pre-treatment with a PI3K/Akt signaling pathway inhibitor (LY294002). Meanwhile, the increase in antioxidant protein expression and antioxidant activity induced by HT was significantly inhibited under the presence of LY294002. Accordingly, inhibiting the PI3K/Akt signaling pathway with LY294002 eliminated the beneficial effect of HT in reducing excessive ROS production. Similarly, Li et al. (2021) found that inhibition of the PI3K/Akt pathway suppressed the activation of Nrf2 by hesperetin and eliminated the reduction in excessive ROS production induced by hesperetin 52. Furthermore, the protective effect of HT against DQ-induced intestinal barrier dysfunction was largely suppressed by LY294002. Collectively, the current research demonstrates that HT provides protection against DQ-induced oxidative stress and ameliorates intestinal damage by activating the PI3K/Akt-Nrf2 pathway.

Mitochondria, as the main energy producers within cells, are not only the major sites of ROS generation but also the main targets of ROS 16. When mitochondria are damaged, they release increased amounts of ROS, further impairing themselves and other cellular organelles, and creating a vicious cycle 58. To reduce oxidative damage in organisms, a crucial self-protective mechanism known as mitophagy has evolved. Mitophagy selectively removes damaged or abnormal mitochondria, preventing them from generating excessive ROS and thereby reducing intracellular oxidative stress 59. Studies have shown that ROS can induce mitophagy by activating the PINK1-Parkin pathway, which clears damaged and redundant mitochondria, playing a critical regulatory role in maintaining mitochondrial health and preventing oxidative stress-related diseases 60, 61. This process plays a crucial regulatory role in maintaining mitochondrial health and preventing oxidative damage-related diseases. In this study, we found that inhibiting the Nrf2 pathway leads to increased expression of mitophagy-related proteins, indicating that mitophagy may participate in the improvement of HT on oxidative stress. Several reports are indicating that plant polyphenols, including HT, curcumin, and resveratrol, can regulate mitophagy to eliminate damaged mitochondria and maintain the intracellular redox balance, thereby improving related diseases 19, 62, 63. In the current research, we observed that DQ treatment triggers the appearance of autophagic vesicles and increases the expression of mitophagy-related proteins *in vitro* and *in vivo*, suggesting that the mitophagy pathway has been initiated. HT treatment can further activate mitophagy, remove damaged mitochondria, and reduce the production of ROS, thereby contributing to the mitigation of oxidative damage. To delve deeper into the role of mitophagy in the improvement of oxidative stress through HT, we conducted experiments using the IPEC-J2 cells oxidative stress model and the mitophagy inhibitor Mdivi-1. It was found that Mdivi-1 pre-treatment decreased the expression of proteins such as PINK1 and Parkin and diminished cell activity, which was consistent with previous data in IPEC-J2 cells 62. Electron microscopy observations also confirmed these findings by showing a decrease in autophagic vesicles in the presence of Mdivi-1. Correspondingly, the antioxidant activity induced by HT was significantly inhibited in the presence of Mdivi-1, leading to an increase in ROS levels. Furthermore, Mdivi-1 eliminates the protective effect of HT against DQ-induced intestinal barrier dysfunction. Similar results have been found in other disease models. A recent study found that α-ketoglutarate promotes mitophagy and inhibits ROS generation to alleviate osteoarthritis, but these effects can be blocked by Mdivi-1 64. Furthermore, Mdivi-1 inhibited the improvement of cardiovascular diseases mediated by traditional Chinese herbal compounds Nuanxinkang and Xinmaikang through PINK1/Parkin-mediated mitophagy 65, 66. These studies demonstrate that mitophagy plays an irreplaceable role in mediating the dietary improvement of oxidative stress-related diseases. In summary, this study establishes that HT promotes mitophagy to reduce the excessive production of ROS, thereby avoiding intestinal damage at the source. This provides a basis for further studying the therapeutic potential of HT in oxidative stress-related diseases.

The Nrf2 signaling pathway and mitophagy reduce ROS levels through different mechanisms to improve oxidative stress. Mitophagy clears damaged mitochondria, reducing the excessive production of ROS at the source, while the Nrf2 pathway promotes the production of antioxidant enzymes to clear excessive ROS. Thus, we hypothesize that there may be interactions between these pathways, but the relationship between them is not yet clear. Interestingly, we also found that inhibiting mitophagy led to increased levels of p-Nrf2 and HO-1 in this study, which might be considered an effective strategy to prevent oxidative damage. That is to say, inhibiting mitophagy leads to increased phosphorylation of Nrf2, and inhibiting the Nrf2 pathway further activates mitophagy in this study. This seems to indicate that there is a negative correlation between the two, jointly regulating ROS levels. When mitophagy is inhibited, the function of mitochondria becomes abnormal or impaired, leading to an increase in ROS production. Although the expression of p-Nrf2 and HO-1 is found to increase, their elevated levels alone may not be sufficient to fully restore mitochondrial function. Consequently, the antioxidant enzymes produced may be inadequate in clearing the excessive ROS, resulting in continuous oxidative damage to intestinal cells. Additionally, the inhibition of mitophagy may also reduce the expression of antioxidant enzymes 62. Autophagosomes, the key sites for protein synthesis and modification, including antioxidant enzymes, are formed during mitophagy 67. The suppression of mitophagy not only reduces the formation and degradation of autophagosomes but may also trigger cellular stress responses, such as endoplasmic reticulum (ER) stress and oxidative stress, which can further affect the expression and activity of antioxidant enzymes. Therefore, when mitophagy is inhibited, we observe a decrease in the activity of antioxidant enzymes, exacerbating the state of oxidative stress within the cells. Similarly, when the Nrf2 signaling pathway is inhibited, although mitophagy increases, intracellular ROS remains at a certain level. This is because normal mitochondria also produce some ROS, which although less than damaged mitochondria. However, the inhibition of the Nrf2 signaling pathway leads to decreased antioxidant enzyme activity, which cannot eliminate ROS, leading to ROS accumulation and still causing intestinal cell damage. Moreover, there may be complex feedback mechanisms or self-regulation of the antioxidant system at play. Recent studies have also found that Nrf2 deficiency can lead to excessive mitophagy induced by PM2.5, which in turn exacerbates mitochondrial damage and worsens respiratory diseases 68. Furthermore, the Nrf2 signaling pathway may directly participate in the regulation of mitophagy 69, 70. Hence, we tend to believe that there is a synergistic relationship between mitophagy and the Nrf2 signaling pathway, which both play an indispensable role in the collaborative regulation of HT's antioxidant effects. So, what would happen if both pathways were inhibited simultaneously? Would it exacerbate oxidative stress, leading to more severe damage? We believe that the Nrf2 signaling pathway and mitophagy are just two major pathways for improving oxidative stress, and there may be other pathways involved, such as immune regulation and programmed cell death. For example, a study has found that HT can prevent dermal papilla cell inflammation under oxidative stress by inducing autophagy 71. However, we will not discuss these pathways here, but further research can explore new pathways by blocking these two pathways. For oxidative stress-related intestinal diseases, we have elucidated that HT improves them by regulating the PI3K/Akt-Nrf2 signaling pathway and mitophagy.

There are several limitations and future perspectives in this study. Firstly, although the pathway inhibitors used in this study were able to inhibit the expression of related proteins, we realize that the possibility of completely suppressing protein expression is limited. Therefore, in the future, gene knockout techniques can be considered to explore this research or seek other more effective treatment methods. Secondly, this study mainly focuses on the potential of HT in the treatment of intestinal oxidative stress diseases, but it does not fully explore its application value in other diseases. Follow-up studies can expand the research scope and investigate the therapeutic effects of HT in other types of diseases, to bring hope to more patients. Additionally, the study is based solely on piglet and cellular models, lacking clinical data support. Future research should include clinical trials to validate the generalizability of the findings. Lastly, exploring the combined application of HT with other antioxidant drugs may achieve better therapeutic effects. This will become an important direction for future research and is expected to provide patients with better treatment options. In a word, this study has made beneficial contributions to the development of the intestinal oxidative stress disease treatment field.

## Conclusions

Current research shows that HT has a protective effect on intestinal oxidative damage, which is mediated by regulating the PI3K/Akt-Nrf2 signaling pathway and mitophagy. Consequently, the consumption of foods rich in HT may be beneficial to intestinal health. The Nrf2 signaling pathway and mitophagy are emerging as novel and effective targets for the treatment of stress-related intestinal diseases. This study opens new avenues for the treatment of intestinal oxidative stress-related diseases and holds the potential to offer more effective therapeutic approaches for patients.

## Supplementary Material

Supplementary figure.

## References

[1] Geertsema S, Bourgonje AR, Fagundes RR, Gacesa R, Weersma RK, van Goor H (2023). The NRF2/Keap1 pathway as a therapeutic target in inflammatory bowel disease. Trends Mol Med.

[2] Grisham MB (1994). Oxidants and free radicals in inflammatory bowel disease. Lancet.

[3] Guo Y, Liu Y, Zhao S, Xu W, Li Y, Zhao P (2021). Oxidative stress-induced FABP5 S-glutathionylation protects against acute lung injury by suppressing inflammation in macrophages. Nat Commun.

[4] Ghelli Luserna di Rora A, Iacobucci I, Martinelli G (2017). The cell cycle checkpoint inhibitors in the treatment of leukemias. J Hematol Oncol.

[5] Dinu M, Pagliai G, Casini A, Sofi F (2018). Mediterranean diet and multiple health outcomes: an umbrella review of meta-analyses of observational studies and randomised trials. Eur J Clin Nutr.

[6] Estruch R, Ros E, Salas-Salvadó J, Covas MI, Corella D, Arós F (2018). Retraction and republication: primary prevention of cardiovascular disease with a mediterranean diet. N Engl J Med 2013;368:1279-90. N Engl J Med.

[7] Estruch R, Ros E, Salas-Salvadó J, Covas MI, Corella D, Arós F (2018). Primary prevention of cardiovascular disease with a mediterranean diet supplemented with extra-virgin olive oil or nuts. N Engl J Med.

[8] Cordaro M, Trovato Salinaro A, Siracusa R, D'Amico R, Impellizzeri D, Scuto M (2021). Hidrox(®) roles in neuroprotection: biochemical links between traumatic brain injury and Alzheimer's disease. Antioxidants (Basel).

[9] Robles-Almazan M, Pulido-Moran M, Moreno-Fernandez J, Ramirez-Tortosa C, Rodriguez-Garcia C, Quiles JL (2018). Hydroxytyrosol: Bioavailability, toxicity, and clinical applications. Food Res Int.

[10] Romana-Souza B, Saguie BO, Pereira de Almeida Nogueira N, Paes M, Dos Santos Valença S, Atella GC (2020). Oleic acid and hydroxytyrosol present in olive oil promote ROS and inflammatory response in normal cultures of murine dermal fibroblasts through the NF-κB and NRF2 pathways. Food Res Int.

[11] Elmaksoud HAA, Motawea MH, Desoky AA, Elharrif MG, Ibrahimi A (2021). Hydroxytyrosol alleviate intestinal inflammation, oxidative stress and apoptosis resulted in ulcerative colitis. Biomed Pharmacother.

[12] Wang Q, Wang CJ, Abdullah, Tian WN, Qiu ZY, Song MY (2022). Hydroxytyrosol alleviates dextran sulfate sodium-induced colitis by modulating inflammatory responses, intestinal barrier, and microbiome. J Agric Food Chem.

[13] Han H, Zhong RQ, Zhang SF, Wang MY, Wen XB, Yi B (2023). Hydroxytyrosol attenuates diquat-induced oxidative stress by activating Nrf2 pathway and modulating colonic microbiota in mice. J Nutr Biochem.

[14] Dröge W (2002). Free radicals in the physiological control of cell function. Physiol Rev.

[15] Liu P, Luo G, Dodson M, Schmidlin CJ, Wei Y, Kerimoglu B (2021). The NRF2-LOC344887 signaling axis suppresses pulmonary fibrosis. Redox Biol.

[16] Wen XB, Tang LX, Zhong RQ, Liu L, Chen L, Zhang HF (2023). Role of mitophagy in regulating intestinal oxidative damage. Antioxidants (Basel).

[17] Sweetman E, Kleffmann T, Edgar C, de Lange M, Vallings R, Tate W (2020). A SWATH-MS analysis of Myalgic Encephalomyelitis/Chronic Fatigue Syndrome peripheral blood mononuclear cell proteomes reveals mitochondrial dysfunction. J Transl Med.

[18] Zhang J, Sun X, Wang L, Wong YK, Lee YM, Zhou C (2018). Artesunate-induced mitophagy alters cellular redox status. Redox Biol.

[19] Dong YZ, Yu MH, Wu YL, Xia T, Wang L, Song K (2022). Hydroxytyrosol promotes the mitochondrial function through activating mitophagy. Antioxidants (Basel).

[20] Dong YZ, Li L, Espe M, Lu KL, Rahimnejad S (2020). Hydroxytyrosol attenuates hepatic fat accumulation via activating mitochondrial biogenesis and autophagy through the AMPK pathway. J Agric Food Chem.

[21] Heinritz SN, Mosenthin R, Weiss E (2013). Use of pigs as a potential model for research into dietary modulation of the human gut microbiota. Nutr Res Rev.

[22] Cao S, Xiao H, Li X, Zhu J, Gao J, Wang L (2021). AMPK-PINK1/Parkin mediated mitophagy is necessary for alleviating oxidative stress-induced intestinal epithelial barrier damage and mitochondrial energy metabolism dysfunction in IPEC-J2. Antioxidants (Basel).

[23] Tian S, Wang J, Yu H, Wang J, Zhu W (2018). Effects of galacto-oligosaccharides on growth and gut function of newborn suckling piglets. J Anim Sci Biotechnol.

[24] Nicholson JK, Holmes E, Kinross J, Burcelin R, Gibson G, Jia W (2012). Host-gut microbiota metabolic interactions. Science.

[25] Hayes JD, Dinkova-Kostova AT (2014). The Nrf2 regulatory network provides an interface between redox and intermediary metabolism. Trends Biochem Sci.

[26] Manna K, Mishra S, Saha M, Mahapatra S, Saha C, Yenge G (2019). Amelioration of diabetic nephropathy using pomegranate peel extract-stabilized gold nanoparticles: assessment of NF-κB and Nrf2 signaling system. Int J Nanomedicine.

[27] Ismail MB, Rajendran P, AbuZahra HM, Veeraraghavan VP (2021). Mangiferin inhibits apoptosis in doxorubicin-induced vascular endothelial cells via the Nrf2 signaling pathway. Int J Mol Sci.

[28] Baek A, Son S, Baek YM, Kim DE (2021). KRT8 (keratin 8) attenuates necrotic cell death by facilitating mitochondrial fission-mediated mitophagy through interaction with PLEC (plectin). Autophagy.

[29] Bhattacharyya A, Chattopadhyay R, Mitra S, Crowe SE (2014). Oxidative stress: an essential factor in the pathogenesis of gastrointestinal mucosal diseases. Physiol Rev.

[30] Moura FA, de Andrade KQ, Dos Santos JCF, Araújo ORP, Goulart MOF (2015). Antioxidant therapy for treatment of inflammatory bowel disease: Does it work?. Redox Biol.

[31] Xiao Y, Huang R, Wang N, Deng Y, Tan B, Yin Y (2022). Ellagic acid alleviates oxidative stress by mediating Nrf2 signaling pathways and protects against paraquat-induced intestinal injury in piglets. Antioxidants (Basel).

[32] Sies H (2017). Hydrogen peroxide as a central redox signaling molecule in physiological oxidative stress: Oxidative eustress. Redox Biol.

[33] Agod Z, Fekete T, Budai MM, Varga A, Szabo A, Moon H (2017). Regulation of type I interferon responses by mitochondria-derived reactive oxygen species in plasmacytoid dendritic cells. Redox Biol.

[34] Kalinichenko AL, Jappy D, Solius GM, Maltsev DI, Bogdanova YA, Mukhametshina LF (2023). Chemogenetic emulation of intraneuronal oxidative stress affects synaptic plasticity. Redox Biol.

[35] Chen Y, Zhang H, Ji S, Jia P, Chen Y, Li Y (2021). Resveratrol and its derivative pterostilbene attenuate oxidative stress-induced intestinal injury by improving mitochondrial redox homeostasis and function via SIRT1 signaling. Free Radic Biol Med.

[36] Wen XB, Wan F, Wu Y, Liu L, Liu YP, Zhong RQ (2023). Caffeic acid supplementation ameliorates intestinal injury by modulating intestinal microbiota in LPS-challenged piglets. Food Funct.

[37] Chen J, Luo Y, Li Y, Chen D, Yu B, He J (2021). Chlorogenic Acid Attenuates Oxidative Stress-Induced Intestinal Epithelium Injury by Co-Regulating the PI3K/Akt and IκBα/NF-κB Signaling. Antioxidants (Basel).

[38] Bertelli M, Kiani AK, Paolacci S, Manara E, Kurti D, Dhuli K (2020). Hydroxytyrosol: A natural compound with promising pharmacological activities. J Biotechnol.

[39] Gavahian M, Mousavi Khaneghah A, Lorenzo JM, Munekata PES, Garcia-Mantrana I, Collado MC (2019). Health benefits of olive oil and its components: Impacts on gut microbiota antioxidant activities, and prevention of noncommunicable diseases. Trends in Food Science & Technology.

[40] Laval L, Martin R, Natividad JN, Chain F, Miquel S, Desclée de Maredsous C (2015). Lactobacillus rhamnosus CNCM I-3690 and the commensal bacterium Faecalibacterium prausnitzii A2-165 exhibit similar protective effects to induced barrier hyper-permeability in mice. Gut Microbes.

[41] Pral LP, Fachi JL, Corrêa RO, Colonna M, Vinolo MAR (2021). Hypoxia and HIF-1 as key regulators of gut microbiota and host interactions. Trends Immunol.

[42] Hwang J, Jin J, Jeon S, Moon SH, Park MY, Yum DY (2020). SOD1 suppresses pro-inflammatory immune responses by protecting against oxidative stress in colitis. Redox Biol.

[43] Zeb A, Choubey V, Gupta R, Kuum M, Safiulina D, Vaarmann A (2021). A novel role of KEAP1/PGAM5 complex: ROS sensor for inducing mitophagy. Redox Biol.

[44] LeBoeuf SE, Wu WL, Karakousi TR, Karadal B, Jackson SR, Davidson SM (2020). Activation of oxidative stress response in cancer generates a druggable dependency on exogenous non-essential amino acids. Cell Metab.

[45] D'Autréaux B, Toledano MB (2007). ROS as signalling molecules: mechanisms that generate specificity in ROS homeostasis. Nat Rev Mol Cell Biol.

[46] Romero-Márquez JM, Navarro-Hortal MD, Jiménez-Trigo V, Muñoz-Ollero P, Forbes-Hernández TY, Esteban-Muñoz A (2022). An olive-derived extract 20% rich in hydroxytyrosol prevents β-amyloid aggregation and oxidative stress, two features of Alzheimer disease, via SKN-1/NRF2 and HSP-16.2 in *Caenorhabditis elegans*. Antioxidants (Basel).

[47] Fusco R, Cordaro M, Siracusa R, Peritore AF, D'Amico R, Licata P (2020). Effects of hydroxytyrosol against lipopolysaccharide-induced inflammation and oxidative stress in bovine mammary epithelial cells: A natural therapeutic tool for bovine mastitis. Antioxidants (Basel).

[48] Illesca P, Valenzuela R, Espinosa A, Echeverría F, Soto-Alarcon S, Ortiz M (2019). Hydroxytyrosol supplementation ameliorates the metabolic disturbances in white adipose tissue from mice fed a high-fat diet through recovery of transcription factors Nrf2, SREBP-1c, PPAR-γ and NF-κB. Biomed Pharmacother.

[49] Xie Y, Shi X, Sheng K, Han G, Li W, Zhao Q (2019). PI3K/Akt signaling transduction pathway, erythropoiesis and glycolysis in hypoxia (Review). Mol Med Rep.

[50] Gorrini C, Gang BP, Bassi C, Wakeham A, Baniasadi SP, Hao Z (2014). Estrogen controls the survival of BRCA1-deficient cells via a PI3K-NRF2-regulated pathway. Proc Natl Acad Sci U S A.

[51] Naguib S, Backstrom JR, Gil M, Calkins DJ, Rex TS (2021). Retinal oxidative stress activates the NRF2/ARE pathway: An early endogenous protective response to ocular hypertension. Redox Biol.

[52] Li J, Wang T, Liu P, Yang F, Wang X, Zheng W (2021). Hesperetin ameliorates hepatic oxidative stress and inflammation via the PI3K/AKT-Nrf2-ARE pathway in oleic acid-induced HepG2 cells and a rat model of high-fat diet-induced NAFLD. Food Funct.

[53] Xiao Q, Piao R, Wang H, Li C, Song L (2018). Orientin-mediated Nrf2/HO-1 signal alleviates H(2)O(2)-induced oxidative damage via induction of JNK and PI3K/AKT activation. Int J Biol Macromol.

[54] Chen JL, Luo YH, Li Y, Chen DW, Yu B, He J (2021). Chlorogenic acid attenuates oxidative stress-induced intestinal epithelium injury by co-regulating the PI3K/Akt and IkappaBalpha/NF-kappaB signaling. Antioxidants (Basel).

[55] Zhang BB, Zeng MN, Li BK, Kan YX, Wang SC, Cao B (2021). Arbutin attenuates LPS-induced acute kidney injury by inhibiting inflammation and apoptosis via the PI3K/Akt/Nrf2 pathway. Phytomedicine.

[56] Li H, Jiang R, Lou L, Jia C, Zou L, Chen M (2022). Formononetin improves the survival of random skin flaps through PI3K/Akt-mediated Nrf2 antioxidant defense system. Front Pharmacol.

[57] Martin MA, Ramos S, Granado-Serrano AB, Rodriguez-Ramiro I, Trujillo M, Bravo L (2010). Hydroxytyrosol induces antioxidant/detoxificant enzymes and Nrf2 translocation via extracellular regulated kinases and phosphatidylinositol-3-kinase/protein kinase B pathways in HepG2 cells. Mol Nutr Food Res.

[58] De Gaetano A, Gibellini L, Zanini G, Nasi M, Cossarizza A, Pinti M (2021). Mitophagy and oxidative stress: The role of aging. Antioxidants (Basel).

[59] Alan P, Vandevoorde KR, Joshi B, Cardoen B, Gao G, Mohammadzadeh Y (2022). Basal Gp78-dependent mitophagy promotes mitochondrial health and limits mitochondrial ROS. Cell Mol Life Sci.

[60] Zhang C, Nie P, Zhou C, Hu Y, Duan S, Gu M (2021). Oxidative stress-induced mitophagy is suppressed by the miR-106b-93-25 cluster in a protective manner. Cell Death Dis.

[61] Wang Y, Nartiss Y, Steipe B, McQuibban GA, Kim PK (2012). ROS-induced mitochondrial depolarization initiates PARK2/PARKIN-dependent mitochondrial degradation by autophagy. Autophagy.

[62] Cao ST, Wang CC, Yan JT, Li X, Wen JS, Hu CH (2020). Curcumin ameliorates oxidative stress-induced intestinal barrier injury and mitochondrial damage by promoting Parkin dependent mitophagy through AMPK-TFEB signal pathway. Free Radic Biol Med.

[63] Koh YC, Ho CT, Pan MH (2023). The role of mitochondria in phytochemically mediated disease amelioration. J Agric Food Chem.

[64] Liu L, Zhang W, Liu T, Tan Y, Chen C, Zhao J (2023). The physiological metabolite α-ketoglutarate ameliorates osteoarthritis by regulating mitophagy and oxidative stress. Redox Biol.

[65] Guan Z, Chen J, Wang L, Hao M, Dong X, Luo T (2023). Nuanxinkang prevents the development of myocardial infarction-induced chronic heart failure by promoting PINK1/Parkin-mediated mitophagy. Phytomedicine.

[66] Cao Y, Chen X, Pan F, Wang M, Zhuang H, Chen J (2023). Xinmaikang-mediated mitophagy attenuates atherosclerosis via the PINK1/Parkin signaling pathway. Phytomedicine.

[67] Lee J, Giordano S, Zhang J (2012). Autophagy, mitochondria and oxidative stress: cross-talk and redox signalling. Biochem J.

[68] Fan X, Dong T, Yan K, Ci X, Peng L (2023). PM2.5 increases susceptibility to acute exacerbation of COPD via NOX4/Nrf2 redox imbalance-mediated mitophagy. Redox Biol.

[69] Song C, Zhang A, Zhang M, Song Y, Huangfu H, Jin S (2023). Nrf2/PINK1-mediated mitophagy induction alleviates sodium fluoride-induced hepatic injury by improving mitochondrial function, oxidative stress, and inflammation. Ecotoxicol Environ Saf.

[70] Gumeni S, Papanagnou ED, Manola MS, Trougakos IP (2021). Nrf2 activation induces mitophagy and reverses Parkin/Pink1 knock down-mediated neuronal and muscle degeneration phenotypes. Cell Death Dis.

[71] Chen Q, Sun T, Wang J, Jia J, Yi YH, Chen YX (2019). Hydroxytyrosol prevents dermal papilla cells inflammation under oxidative stress by inducing autophagy. J Biochem Mol Toxicol.

