# Supplementary material for: The PI3K/Akt-Nrf2 Signaling Pathway and Mitophagy Synergistically Mediate Hydroxytyrosol to Alleviate Intestinal Oxidative Damage
Source: Int J Biol Sci. 2024 Aug 6;20(11):4258–76. doi: 10.7150/ijbs.97263 (PMC11379072; doi:10.7150/ijbs.97263)
Supplement: Supplementary file 1 — Supplementary figure. [file ijbsv20p4258s1.pdf]

The PI3K/Akt-Nrf2 Signaling Pathway and Mitophagy Synergistically Mediate Hydroxytyrosol to Alleviate Intestinal Oxidative Damage

Xiaobin Wen[#], Shanlong Tang[#], Fan Wan, Ruqing Zhong, Liang Chen[*], Hongfu Zhang[*]

State Key Laboratory of Animal Nutrition and Feeding, Institute of Animal Sciences, Chinese Academy of Agricultural Sciences, Beijing, 100193, China.

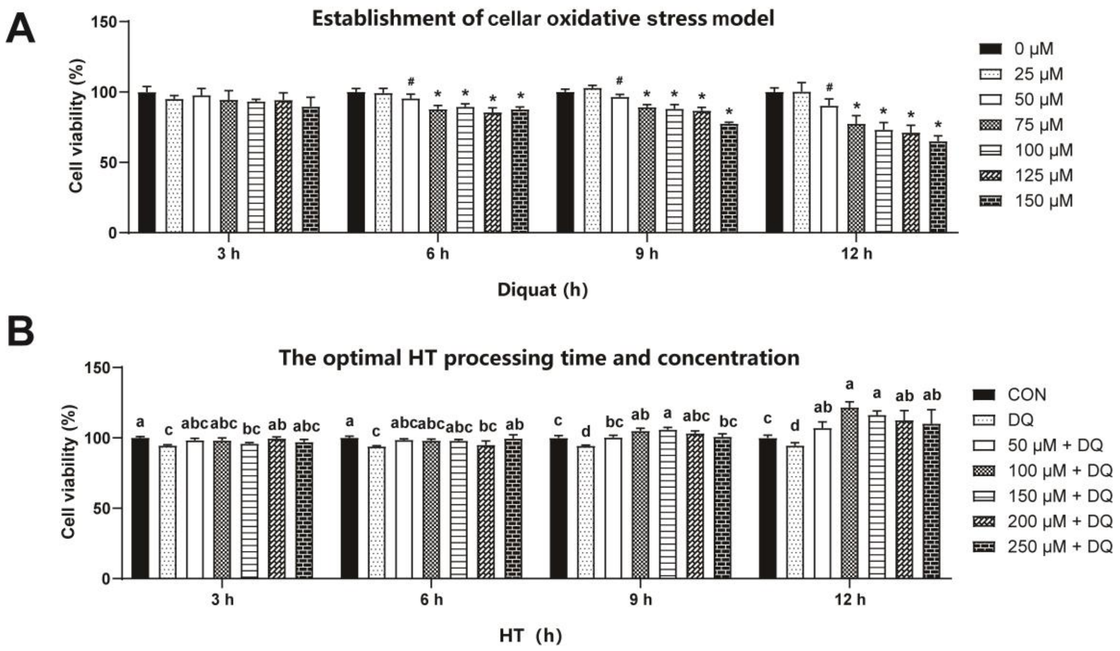

Fig. S1 Establishment of oxidative stress model in IPEC-J2 cells. (A) The effect of incubation duration and concentration of DQ on IPEC-J2 cell viability was determined by CCK-8 assay. (B) The effect of incubation duration and concentration of hydroxytyrosol (HT) on the viability of IPEC-J2 cells pretreated with DQ was determined by CCK-8 assay. Values are means ± SE. Different letters and * represent significant differences ($P < 0.05$), # represent a trend ($0.05 < P < 0.01$).
